# Early Identification of Exacerbations in Patients with Chronic Obstructive Pulmonary Disease (COPD)

**DOI:** 10.3390/jcm14020397

**Published:** 2025-01-10

**Authors:** Ilektra Voulgareli, Elvira-Markela Antonogiannaki, Konstantinos Bartziokas, Stavrina Zaneli, Petros Bakakos, Stelios Loukides, Andriana I. Papaioannou

**Affiliations:** 1 2nd Respiratory Medicine Department, “Attikon” University Hospital, National and Kapodistrian University of Athens Medical School, 12462 Athens, Greece; ilektravoul@gmail.com (I.V.); kantonogiannaki@gmail.com (E.-M.A.); loukstel@med.uoa.gr (S.L.); 2 Independent Researcher, 42131 Trikala, Greece; 3 1st Respiratory Medicine Department, “Sotiria” Chest Hospital, National and Kapodistrian University of Athens Medical School, 11527 Athens, Greece; stavzaneli@gmail.com (S.Z.); petros44@hotmail.com (P.B.); papaioannouandriana@gmail.com (A.I.P.)

**Keywords:** COPD, exacerbations, self-management intervention, telehealth, biomarkers

## Abstract

Exacerbations of Chronic Obstructive Pulmonary Disease (COPD) have a substantial effect on overall disease management, health system costs, and patient outcomes. However, exacerbations are often underdiagnosed or recognized with great delay due to several factors such as patients’ inability to differentiate between acute episodes and symptom fluctuations, delays in seeking medical assistance, and disparities in dyspnea perception. Self-management intervention plans, telehealth and smartphone-based programs provide educational material, counseling, virtual hospitals and telerehabilitation, and help COPD patients to identify exacerbations early. Moreover, biomarkers such as blood eosinophil count, fibrinogen, CRP, Serum amyloid A(SAA),together with imaging parameters such as the pulmonary artery-to-aorta diameter ratio, have emerged as potential predictors of exacerbations, yet their clinical utility is limited by variability and lack of specificity. In this review, we provide information regarding the importance of the early identification of exacerbation events in COPD patients and the available methods which can be used for this purpose.

## 1. Introduction

Chronic Obstructive Pulmonary Disease (COPD) is characterized by persistent respiratory symptoms and airflow limitation due to airway and/or alveolar abnormalities, usually provoked by significant exposure to noxious particles or gases [1]. COPD is an underdiagnosed condition and almost 65 million people worldwide are thought to have COPD. By 2030, COPD is anticipated to overtake heart disease as the third major cause of death globally. Chronic cough, unusual sputum output, and dyspnea with exertion are typical symptoms of COPD. COPD exacerbations are defined as events characterized by dyspnea and/or cough and sputum that worsen over 14 days and may be accompanied by tachypnea and/or tachycardia, and they are associated with increased local and systemic inflammation caused by airway infection, pollution, or other insult to the airways, which occurs more frequently as lung function deteriorates, marking the gradual progression of COPD [2]. Exacerbations vary in their severity from temporary functional status worsening to lethal incidents [1]. Lung function and quality of life often rapidly decline in association with recurrent exacerbations [3,4], and severe exacerbations are linked to a higher mortality rate [5]. Some patients never reach their pre-exacerbation state of health [6]. Several studies have found a significant rate of recurrence [7] and mortality rate [8] in those who experience severe COPD exacerbations that necessitate hospitalization. However, despite the importance of early recognition, most COPD patients seek medical help several days after the exacerbation onset [9], which has also been found to be associated with adverse outcomes such as longer time to recovery, worse health-related quality of life (HRQoL), and more frequent need for hospital admission [10]. Hence, the early recognition of COPD exacerbation is essential both for patients and health care providers.

## 2. Definition and Severity of COPD Exacerbations

COPD exacerbations are heterogeneous events with varying pathogeneses, etiologies, symptoms, frequency, severity, and biomarkers. The 2024 GOLD states that the exacerbation of COPD is “an event characterized by dyspnea and/or cough and sputum that worsen over <14 days” [1]. Based on the timing of the events and clinical symptoms (typical symptoms include increased dyspnea, cough, increased sputum volume, and/or purulent sputum), this definition is suitable for clinical purposes. COPD exacerbations can be triggered by various microorganisms and inflammatory factors [1]. Key bacterial pathogens, such as Haemophilus influenzae, Streptococcus pneumoniae, Moraxella catarrhalis, and Pseudomonas aeruginosa, are commonly involved, with viral infections like influenza, rhinovirus, respiratory syncytial virus (RSV), and coronavirus also playing a role [1]. Moreover, chronic airway inflammation is often exacerbated by environmental factors like air pollution, allergens, or cold air [1].

The risk of experiencing a COPD exacerbation is linked to several factors (Table 1), including a history of at least one prior exacerbation in the past 12 months, more severe COPD (as determined by FEV1), the presence of chronic bronchitis, and other comorbid conditions (such as gastroesophageal reflux or pulmonary hypertension) [11]. Additionally, a pulmonary artery-to-aorta ratio greater than one on computed tomography (CT) imaging has been identified as an independent risk factor for future COPD exacerbations [12].

Especially as far as comorbidities are concerned, over 80% of COPD patients have at least one chronic comorbidity, which negatively impacts quality of life, exacerbation risk and survival [14]. The Charlson Comorbidity Index (CCI), introduced in 1987, is a recognized independent risk factor for exacerbations and mortality [15]. Higher CCI scores correlate with reduced survival; patients with scores ≥ 5 (indicating ≥4 comorbidities) face a mortality rate over five times higher than those without comorbidities [16]. Increased comorbidities also significantly raise the risk of hospital readmission and longer stays, with each additional condition increasing readmission risk by 47% [17]. COPD comorbidities primarily affect the cardiovascular, respiratory, mental and endocrine systems and include conditions like cardiovascular disease, pulmonary hypertension, lung cancer, diabetes, osteoporosis and anxiety or depression [14]. Cardiovascular diseases are the most common and pose the highest risk among these comorbidities [18].

Furthermore, it has to be mentioned that the presence of bronchectasis in COPD patients is associated with more frequent and severe exacerbations, prolonged episodes, increased bacterial colonization and heightened inflammation, leading to higher hospitalization and mortality risks [19]. This coexistence represents a distinct phenotype with a poorer prognosis, highlighting the need for thorough evaluation and tailored management [19].

Finally, smoking cessation is crucial in reducing the risk of COPD exacerbations. Studies have demonstrated that quitting smoking leads to a decreased risk of exacerbations, with the reduction being more pronounced the longer a patient remains abstinent. For instance, individuals who quit smoking for over ten years exhibit a significantly lower risk of COPD exacerbations compared to current smokers [20].

The diagnostic approach to an exacerbation involves a complete and thorough clinical assessment of the patient for evidence of COPD exacerbation, and potential respiratory and non-respiratory concomitant diseases should be explored [13]. This assessment should include symptoms (severity of dyspnea as measured by a visual analog scale and documentation of the presence of cough) and signs (tachypnea, tachycardia, sputum color and volume, accessory muscle use, bronchospasm) [13]. Determination of the severity of an exacerbation should include other appropriate investigations including pulse oximetry, complete blood count, brain natriuretic peptide (BNP), C-reactive protein (CRP) and/or arterial blood gas (ABG) analysis [13].

Short-acting bronchodilators, including β2 agonists and muscarinic antagonists, are the cornerstone of exacerbation treatment [1]. These can be delivered via a metered-dose inhaler or nebulizer, with the choice depending on the severity of the exacerbation and the patient’s ability to use the device [1]. Nebulizers are preferred for those unable to use handheld devices effectively [1]. Furthermore, oral glucocorticoids are recommended initially [1]. For hospitalized patients unable to take oral medications, intravenous methylprednisolone (40 mg daily) is advised, whereas the role of inhaled corticosteroids during acute exacerbations remains unstudied [1]. As far as antibiotics are concerned, they are indicated for patients with bacterial infections or severe symptoms, such as increased cough, sputum volume, or purulence [13]. Common options include amoxicillin–clavulanate, macrolides, or tetracyclines. Fluoroquinolones are recommended for suspected *Pseudomonas* infections [13]. If no improvement occurs within 72 h, reassessment and sputum culture are necessary. Also, chest physiotherapy and airway clearance techniques, in combination with smoking cessation can help in the management of a COPD exacerbation [1]. Finally, pulmonary rehabilitation, a comprehensive program that combines physical exercise, education and behavioral therapy, plays a key role in enhancing lung function, reducing symptoms and preventing future exacerbations [1].

Currently, exacerbations are classified as mild, if they are not related with very severe dyspnea, hypoxemia and tachypnea or tachycardia, moderate if tachypnea and/or tachycardia together with respiratory failure are present but without respiratory acidosis, and severe for those with all the above in the presence of respiratory acidosis [1]. Usually, mild exacerbations are treated only with short-acting bronchodilators (SABDs), while in moderate exacerbations, systemic corticosteroids ± antibiotics in addition to SABs are needed. Finally, COPD patients experiencing severe exacerbations require treatment in the emergency department or hospital admission [1].

Diagnosis of an exacerbation of COPD is based on the patient’s medical history in combination with clinical and laboratory findings. However, most of the symptoms of a COPD exacerbation are also present in numerous diseases (both pulmonary and non-pulmonary), making the existing definition lack specificity and sensitivity [21]. In the meantime, most patients are unable to recognize the very early warning signs of an exacerbation and to differentiate their worsening symptoms from daily fluctuations. Consequently, specialists argue that a revised definition is required, which will incorporate validated biomarkers and classify particular symptoms over time [21].

The perception of patients in the recognition of COPD exacerbations is also controversial, making the distinction between COPD exacerbations and fluctuation in COPD symptoms hard. Most studies determine worsening that lasts for at least 2 days and will also require at least 5 consecutive days before returning to the baseline level as an exacerbation rather than a day-to-day variation in symptoms. Finally, there is no worldwide agreement or standardized definition of how the start and end points of exacerbations should be defined, despite the availability of guidelines and attempts to clarify best practices [21].

## 3. Underdiagnosing COPD Exacerbations

COPD patients do not always seek medical care when they develop symptoms indicative of the onset of an acute exacerbation. It has been discovered that while patients seldom ever report colds or wheezing, they frequently report an exacerbation when they cough and their sputum volume changes [22]. Interestingly, COPD patients with lower FEV_1_ are more likely to seek medical assistance, although older individuals are less likely to report an exacerbation [22]. Moreover, patients, especially the older ones, often find it challenging to understand the difference between a COPD exacerbation and daily fluctuation of baseline symptoms [23], resulting in a delay to ask for medical care [24,25]. However, according to other studies, COPD patients who experience frequent exacerbations, and especially those who had at least one treated exacerbation the preceding year, seem to be more able to detect a new exacerbation event [26]. These individuals would rather wait and see if their symptoms improve with home-based treatments [26]. On top of that, dyspnea perception seems to be a crucial factor in the identification of a COPD exacerbation since it appears to be amplified in frequent exacerbators and attenuated in infrequent exacerbators, possibly as a result of central dyspnea perception bias [27]. This finding may help to explain why those with poor perception are less likely to ask for medical treatment [27]. Patients with COPD who are reluctant to seek medical help are more likely to be admitted to the hospital [28] possibly because as the exacerbation worsens, bronchodilator responsiveness may deteriorate [28]. Furthermore, patients who delay seeking medical help seem to be more likely to experience a respiratory infection that worsens and results in more severe exacerbation [9]. While roughly one-third of all exacerbations occur within 8 weeks, despite complete recovery from the preceding episode, it is also assumed that the most significant risk factor for a new exacerbation is a previous exacerbation [7]. It has been reported [29] that patients who are hospitalized for longer than 7 days and those whose FEV_1_ on the day of admission is reduced by more than 20% from stable-state values are more likely to experience a subsequent COPD exacerbation.

## 4. The Importance of Early Identification of a COPD Exacerbation

It has been estimated that a large proportion of COPD exacerbations are not reported by the patients, although even these unreported COPD exacerbations are associated with all the already known negative long-term adverse outcomes [30,31,32]. In a previous study including a cohort of 73,106 patients hospitalized for the first time for a COPD exacerbation, it was found that every exacerbation increases the risk of subsequent exacerbations, while the time interval between the next severe exacerbations is shortened [33]. In order to prevent the progress of an exacerbation, early recognition and detection and early initiation of pharmacological treatment are crucial. The sooner treatment is initiated after the diagnosis of a COPD exacerbation, the earlier the symptoms will resolve, the quality of life will improve and the risk of hospitalization will minimize [34].

In a previous study, Makris et al. [35] reported that the rate of FEV_1_ decline was higher in frequent exacerbators, with a mean decline of 74 mL/year, while Donaldson et al. [3] also observed a faster decline in FEV_1_ in frequent exacerbators compared to infrequent exacerbators. Notably, another significant issue is how COPD exacerbations affect the patients’ survival. According to a study by Van Hirtum et al. [36], 15-year survival for hospitalized COPD patients was found to be 82% lower compared to the general population, indicating that survival after hospitalization for a COPD exacerbation is reduced. Furthermore, in a study including 1824 COPD patients, it was observed that prior hospital admission for acute exacerbation was an independent risk factor for death [28]. Previous research from Wilkinson et al. [10] has also shown that COPD patients who do not receive treatment for exacerbations have worse health-related quality of life and may contribute to increased morbidity from these episodes, so early recognition and access to therapy for an exacerbation are essential [10]. Another important finding is that patients with moderate or severe exacerbations appear to have greater COPD-related health care costs overall [37]. Pasquale et al. [37] have shown that health care utilization and costs increase in a direct proportion to the severity of the condition, supporting the hypothesis that the reduction of [37] two or more exacerbations to none might actually results in annual savings of USD 5125 for COPD-related health care utilization and USD 11,599 for total health care utilization per patient. Moreover, eliminating exacerbations would result in annual savings of approximately USD 13,296 per patient, whereas even lowering the severity of exacerbations from severe to moderate would result in annual savings of approximately USD 9409 [37] per patient. Similarly, Yu et al. [38] found that the severity of COPD exacerbations correlated with an increase in health care costs and showed that all-cause expenditures for patients who experienced any exacerbation were twice as high as they were for individuals who did not have any exacerbation. In conclusion, any COPD exacerbation not only is a risk factor for future exacerbations and negatively influences lung function and survival, but it also results in increased costs for the health care system, making, once more, exacerbation prevention essential.

## 5. Self–Management Intervention

A self-management intervention of COPD is a structured plan of disease management which, however, is personalized according to each patient’s needs. Furthermore, this plan is often multi-component and includes different goals of motivating, engaging and supporting the patients to positively adapt their health behavior(s) and to develop skills which will result in a better management of their disease [39]. The ultimate goals of self-management are as follows: (a) optimization and preservation of physical health; (b) reduction in symptoms and functional impairment in daily life and increase in emotional well-being, social well-being and quality of life; and (c) establishing effective alliances with health care professionals, family, friends and community [39]. The administration of personalized action plans to be used when the patient has symptoms consistent with an exacerbation is very important since it has been shown that they lead to improvements of quality of life as measured by the SGRQ and decrease the likelihood of respiratory-related hospital admissions [40]. However, it seems that these self-management plans do not lower all-cause mortality risk [40]. Jonkman et al. [41] confirmed the above observations through a systematic literature search, which included fourteen trials, and concluded that self-management programs improve COPD patients’ health-related quality of life and increase the time to the first respiratory-related hospitalization, but do not have any impact on mortality. Likewise, Bourbeau et al. [42] found that patients with advanced COPD who participated in a self-education program had fewer hospital admissions for COPD exacerbations as well as for any cause, according to a multicenter trial that included patients with advanced COPD and at least one hospital admission for an exacerbation in the previous year. A self-management plan that includes education sessions, a thorough written action plan for self-treatment of exacerbations, or monthly follow-up calls from a health professional may all help minimize emergency department visits [43]. Moreover, a written action plan could enhance recovery from a COPD exacerbation [44]. To ensure that the positive effects continue after the programs are over, it is thought that self-management treatments should focus on long-term behavioral change [45]. Hence, self-management programs should also include treatments that are successful in bringing about behavior change and ensuring maintenance [45]. Cognitive behavioral therapy is a significant therapeutic approach that can be utilized to affect behavioral changes [45]. The goal of cognitive behavioral therapy is to help patients recognize the different types, impacts, and interactions of thoughts, interpretations, and presented symptoms, feeling states, and behavior in relation to specific problem areas [45]. It is an organized and time-limited psychological intervention [45]. However, it should be always kept in mind which patients are more likely to follow a self-management plan and benefit the most. Health care professionals may have to choose the appropriate patient to implement self-management programs, and it seems that factors like influenza vaccination, cardiac comorbidity, younger age or lower FEV_1_ seem to be related to compliance with written action plans [44]. On the other hand, Fan et al. [46] ended their study sooner because they found that COPD patients who were given a self-management plan had higher mortality compared to those in the control group, a fact which was inconsistent with other trials. It is unknown whether variations in program characteristics can account for the variety in effects of self-management interventions in patients with COPD [47]. The aim of a previous meta-analysis including 14 randomized controlled trials (3282 patients) was to determine the types of COPD self-management treatments which are more beneficial [47]. The authors concluded that the chance of being hospitalized for any reason decreased with each additional month of the self-management intervention [47]. Planning interventions that are most effective should use “if-then” plans, taking into consideration important and pertinent cues, providing examples of signals, being directed rather than user-defined, and incorporating boosters [48]. Thus, it is crucial that further multicenter trials are conducted to describe the phenotypes that will benefit the most.

## 6. Telehealth/Telemonitoring and Smartphones

Even if an exacerbation is not severe enough to require a visit to the emergency department and/or hospital admission, it might have a substantial impact on the patient’s health. Telehealth and telemonitoring can assist patients and doctors to identify symptom changes [49]. The terms “telehealth” and “telemedicine” are similar, and describe more extensive interventions than telemonitoring [50] since they also can include education, counseling, virtual hospitals, self-management training, or telerehabilitation in addition to telemonitoring [49]. However, the choice between telehealth platforms and smartphone-based solutions remains context-dependent, as each has distinct advantages. Telehealth platforms connected to hospitals and clinics provide seamless access to electronic health records and diagnostic tools, offering comprehensive care that may not always be achievable through smartphones [51]. These platforms, often accessed via computers or tablets, enable providers to simultaneously view patient records, diagnostic images and conduct consultations, enhancing the depth of clinical interactions [51]. On the other hand, compared to smartphones, telehealth systems lack portability, making them less convenient for patients on the move, and they often require substantial investments in infrastructure, driving up costs [52]. In contrast, smartphones are widely owned, easier to use for diverse populations, and generally more affordable [52].

In a multicenter international trial named COMET (COPD Patient Management European Trial) [53], patients were offered special education and coaching based on the “Living Well with COPD” self-management program (www.livingwellwithcopd.com) [42] and were followed up by medical experts with competence in home-based therapies and chronic patient care. Patients actively provided updates on their health status when their symptoms worsened or at least once a week [53]. The data were automatically gathered by an e-health website portal, which noted whether this information was favorable, unfavorable, or alarming [46]. The patient was contacted by case managers to inquire about their symptoms, each time they had symptom deterioration or the online portal received an alarm status [46]. The group which followed this telemonitoring plan had fewer acute care hospitalization days per year, a lower BODE (body mass index, airflow obstruction, dyspnea, and exercise) index, and both decreased incidence of exacerbations and mortality rate [53]. Moreover, Claxton et al. [54] developed a smartphone-based system for speedy and accurate COPD exacerbation identification. The algorithm properly identified the existence or absence of an exacerbation based on patient-reported parameters (such as age, fever, and the onset of a new cough) and audio data from five coughs [54]. A systematic review and meta-analysis of 22 studies [49] examined the effectiveness of a telemonitoring strategy to reduce or eliminate severe COPD exacerbations. The study’s final finding was that telemonitoring may reduce unnecessary visits to the emergency department, but it is unlikely to decrease the number of hospitalizations for COPD exacerbations [44]. Also, it was shown that COPD patients much valued telemonitoring and that it could be easily implemented into their present care [49]. Similarly, another meta-analysis of six studies which compared the effectiveness of smartphone treatments in reducing COPD exacerbations to standard therapy showed that smartphones can be used for COPD exacerbation reduction [55]. On the other hand, in another trial named Project on Managing Chronic Obstructive Pulmonary Disease with Remote Patient Management (PROMETE II), although COPD patients were instructed to actively monitor their blood pressure, oxygen saturation, heart rate, and spirometry at home, while a device connected to their primary oxygen source passively recorded data on respiratory rate (and adherence to oxygen therapy), no decrease in emergency visits or hospital admissions for COPD-related events was observed over a 12-month period [56]. Similar were the results of another study [57], in which a COPD telemonitoring service was used to analyze the symptom scores and physiological parameters including FEV_1_, pulse rate, and SpO_2_, concluding that physiological indicators were unable to differentiate between exacerbations and isolated bad days [57]. On the contrary, a review [58] which included two comparative observational studies and seventeen randomized controlled trials indicated that regular physician evaluation through remote home monitoring could contribute to a decrease in COPD-related hospital admissions. According to the above information, one could come to the conclusion that the usefulness of telehealth and smartphones has not been yet proven and results of existing trials should be evaluated carefully, given the variability of care settings and the low-quality research.

## 7. Biomarkers for Predicting a COPD Exacerbation

As mentioned above, COPD exacerbations are important indicators of future risk and can lead to quality-of-life deterioration, increased hospitalization rates and increased mortality. The symptoms that patients usually complain about can vary a lot from day to day and from person to person. The use of biomarkers for predicting which patient will develop a COPD exacerbation is a very important goal for the medical community. The biomarkers are shown in Table 2.

Liu et al. [59] studied different hemogram indexes (platelet–lymphocyte ratio (PLR), platelet × neutrophil/lymphocyte ratio [systemic immune-inflammation index (SII)], and monocyte × neutrophil/lymphocyte ratio [systemic inflammation response index (SIRI)]) in COPD patients presenting with an exacerbation and found that combining the most important inflammatory hemogram index (PLR) with other indexes was a promising predictor of exacerbation in patients with stable COPD [59].

Similarly, it has been reported that high levels of certain T2 biomarkers in the airways (like FeNO ≥ 20 ppb, blood eosinophil count ≥ 300 cells/mm or both) are associated with an increased risk of moderate or severe COPD exacerbations, and that combining FeNO with blood eosinophil count can make the predictions even more accurate [60]. This suggests that some of the COPD exacerbations are related to an increase in T2 activity in the airways [60].

The use of blood eosinophil count as an exacerbation predictor was also confirmed by the Copenhagen General Population Study [61], which declared that COPD patients with blood eosinophils greater than or equal to 0.34 × 10^9^ cells per liter were more likely to experience both severe and moderate exacerbations. Similarly, the COPD Gene study found that blood EOS count ≥ 300/μL increased the risk of COPD exacerbations by 1.32 times [62]. However, it is worth mentioning that opposite results were reported from another trial in which it was reported that patients with low blood eosinophil levels (<150 cells/μL) were more likely to experience a COPD exacerbation [63].

Patients with stable COPD were more likely to experience frequent exacerbations [64] if CRP, fibrinogen, and leukocyte levels were all elevated at the same time, and this was also observed in patients with milder COPD and those who had never experienced COPD exacerbations [64]. The above findings were also confirmed in a different trial [65] in which white blood cell (WBC) count and levels of CRP, IL-6, IL-8, fibrinogen, and TNF-a were measured in peripheral blood in 1755 COPD patients, showing that the exacerbation frequency was substantially higher in patients who were persistently inflamed than in those who were not.

Respiratory viruses, bacteria, and other infections are the primary causes of COPD exacerbation occurrence. The most studied non-specific acute-phase protein in COPD exacerbations is C-reactive protein (CRP) [66], which can serve as a biomarker for infection. However, CRP’s limited utility stems from the fact that it is not disease-specific and is increased in both bacterial and viral infections. Specific biomarkers of bacterial infection, such as procalcitonin (PCT) and CD64, are significant diagnostic and therapeutic indicators for the administration of antibiotics to patients with COPD exacerbations [67,68]. Quint et al. [69] also demonstrated that plasma concentrations of interferon-inducible protein-10 (IP-10) were significantly higher in patients who were tested positive for the rhinovirus and experienced an acute COPD exacerbation compared to patients who tested negative. These findings raise the possibility that elevated serum IP-10 levels could serve as a biomarker of rhinovirus infection.

Soluble urokinase-type plasminogen activator receptor (suPAR) is a soluble form of the urokinase plasminogen activator receptor (uPAR), which can be found in various body fluids, including blood, urine, and cerebrospinal fluid, and is positively correlated with the activation of the immune system [70]. Earlier studies have shown that serum suPAR may reflect the inflammatory process in COPD, and this increase may be particularly effective for patients with severe and very severe airflow obstruction [70,71]. A meta-analysis evaluated its clinical use in COPD and found that suPAR level was higher in during COPD exacerbations and decreased after treatment [72], and thus has the potential to be used for their early identification.

According to Pazarli et al. [73], patients with varying severities of COPD exacerbations had significantly different serum PCT levels. The sensitivity and specificity of the PCT in differentiating between mild and moderate COPD exacerbation were 82% and 91%, respectively, using a PCT cutoff value of 0.07 ng/mL. Patel et al. [74] also showed that CRP and PCT levels in saliva of patients with COPD exacerbations were substantially elevated, while there was a strong correlation between the blood and salivary levels of CRP. The authors expressed the hypothesis that the assessment of CRP in saliva may be a non-invasive method to estimate the exacerbation severity. Moreover, in a prospective cohort study [64], it has been reported that CRH at a cutoff point of 3 mg/L white blood cells (9 × 10^9^/L), and fibrinogen (14 µmol/L) were predictive factors of a five-year increased risk of exacerbations. Indeed, patients with a previous COPD classification of C to D had a higher exacerbation rate compared to patients without high biomarker levels (i.e., 62% vs. 24%, respectively). Longer hospital stays were also related with higher PCT and CRP values.

Serum amyloid A (SAA) is an acute-phase protein, induced, like CRP, by inflammatory mediators, including IL-6, IL-1b, and TNF-a, that rise acutely during COPD exacerbations [75]. Through stimulation of inflammatory factors, SAA can rise quickly, peaking in 8–12 h, and then quickly return to normal once the inflammation is under control. In COPD patients from the Melbourne Longitudinal COPD Cohort (an open cohort of participants with moderate to severe COPD GOLD stages II–IV), both SAA and C-reactive protein (CRP) were elevated at the exacerbation onset compared with stable disease while SAA was a more sensitive and specific inflammatory biomarker compared to CRP during exacerbations [76]. Additionally, it has been demonstrated that SAA has a crucial role in determining the COPD exacerbation severity and the need for hospitalization.

Fibrinogen is a critical modulator of tissue damage, fibrosis formation, and inflammation and has been employed as a biomarker of COPD severity evaluation. Increased fibrinogen levels at baseline have been related with increased incidence of exacerbations, hospitalizations and all-cause mortality and were associated with COPD severity [77,78]. According to a previous study, the levels of fibrinogen increased during exacerbations and returned back to normal 40 days later [77], whereas another study has shown that the levels of circulating fibrinogen can be used to evaluate the exacerbation severity, while fibrinogen levels > 3.55 g/L in patients treated with non-invasive positive-pressure ventilation (NPPV) could independently predict NPPV failure.

The U.S. Food and Drug Administration qualified plasma fibrinogen as a biomarker in the context of use as an enrichment tool for recruitment into clinical trials after a combined analysis of five cohorts, including the ECLIPSE (Evaluation of COPD Longitudinally to Identify Predictive Surrogate Endpoints) cohort, revealed that using a clinical history of exacerbations increased the ability to predict the occurrence of future events, though not their nature [79]. However, adding plasma fibrinogen to clinical trial use has a negligible added value and lacks the sensitivity and specificity to be applied individually in clinical practice [80]. In addition, a study of data from the COPD Gene and ECLIPSE trials revealed the existence of a precise imaging biomarker [12]. A threefold increase in the likelihood of exacerbations was independently linked to the pulmonary artery-to-aorta diameter ratio which was indicative of pulmonary hypertension when it exceeded the value of 1 [12]. Physical indicators, such as elevated respiratory impedance, were also prognostic of subsequent exacerbations [81]. Eventually, studies highlight the significance of using RV:TLC as a marker for lung hyperinflation, which serves as a strong prognostic indicator for mortality and exacerbations in COPD patients [82]. Research by Kim YW et al. [83], using RV:TLC, examined the effects of resting hyperinflation on exacerbations and mortality in 310 COPD patients over an average follow-up of approximately five years. Resting hyperinflation was found to independently predict earlier and more frequent exacerbations as well as increased mortality risk.

Finding a reliable biomarker for COPD exacerbations has been difficult for many years. Yet, it appears that most people agree that this “global” biomarker is probably not present because of the variability of exacerbations [84,85].

## 8. Conclusions

COPD patients frequently experience acute exacerbations, which is linked to both an increase in the need for medical services and a serious decline in lung function. Early identification of COPD exacerbations by incorporating self-management intervention, telehealth and the use of smartphones into clinical care may help patients control their own symptoms, enhance their quality of life and reduce exacerbation rate. Self-management strategies equip patients with the tools to track their symptoms closely and respond promptly to changes in their condition. At the same time, the rapid progress in telehealth has introduced new possibilities for remote monitoring, enabling health care providers to identify early indicators of exacerbations and address them before they worsen. Additionally, the widespread availability of smartphones has paved the way for the creation of intuitive apps and tools designed specifically for COPD care. These digital platforms support real-time interaction between patients and health care professionals, deliver tailored health insights and promote consistent adherence to treatment regimens.

## Figures and Tables

**Table 1:** Factors linked to an increased risk of a COPD exacerbation.

History of at least one prior exacerbation in the past 12 months More severe COPD (FEV_1_ ≤ 30%) Presence of chronic bronchitis Presence of bronchiectasis Presence of gastroesophageal reflux Pulmonary hypertension Pulmonary artery-to-aorta ratio > 1 on computed tomography (CT) Female sex

Modified from Carlin BW. Exacerbations of COPD. Respir Care. 2023 Jul;68(7):961-972. doi: 10.4187/respcare.10782. PMID: 37353338; PMCID: PMC10289624 [13].

**Table 2:** Biomarkers for predicting a COPD exacerbation.

Hemogram indexes	Platelet–lymphocyte ratio (PLR) Platelet × neutrophil/lymphocyte ratio Monocyte × neutrophil/lymphocyte ratio
T2 biomarkers	FeNO ≥ 20 ppb and/or Blood eosinophil count ≥300 cells/mm
Inflammatory markers	CRP Fibrinogen White blood cells (WBCs) IL-1b, IL-6, IL-8, TNF-a Procalcitonin (PCT), CD64 Interferon-inducible protein-10 (IP-10) Soluble urokinase-type plasminogen activator receptor (suPAR) Serum amyloid A (SAA)
Other biomarkers	Pulmonary artery-to-aorta diameter ratio Respiratory impedance RV:TLC
